# Determination of Multiclass Antibiotics in Fish Muscle Using a QuEChERS-UHPLC-MS/MS Method

**DOI:** 10.3390/foods13071081

**Published:** 2024-04-01

**Authors:** Yousra Aissaoui, Gabriel Jiménez-Skrzypek, Javier González-Sálamo, Malika Trabelsi-Ayadi, Ibtissem Ghorbel-Abid, Javier Hernández-Borges

**Affiliations:** 1 Useful Materials Laboratory (LMU), National Institute for Physical and Chemical Research and Analysis (INRAP), Ariana 2020, Tunisia; yousra.issaoui@fsb.ucar.tn; 2 Departamento de Química, Unidad Departamental de Química Analítica, Facultad de Ciencias, Universidad de La Laguna (ULL), Avda. Astrofísico Fco. Sánchez, s/n, 38206 San Cristóbal de La Laguna, Spain; gjimenez@ull.edu.es (G.J.-S.); jgsalamo@ull.edu.es (J.G.-S.); 3 Instituto Universitario de Enfermedades Tropicales y Salud Pública de Canarias, Universidad de La Laguna (ULL), Avda. Astrofísico Fco. Sánchez, s/n, 38206 San Cristóbal de La Laguna, Spain; 4 Laboratory of Application Chemistry to the Resources and Natural Substances and the Environment (LACReSNE), Faculty of Science of Bizerte, University of Carthage, Zarzouna, Bizerte 7021, Tunisia; malika.trabelsiayadi@gmail.com

**Keywords:** antibiotics, quinolones, QuEChERS, farmed fish, mass spectrometry

## Abstract

The surging global demand for fish has increased aquaculture practices, where antibiotics have become indispensable to prevent diseases. However, the passive incorporation of these compounds into the diet may have adverse effects on human health. In this work, the QuEChERS method combined with ultra-high-performance liquid chromatography tandem mass spectrometry was applied for the determination of 10 multiclass antibiotics (5 quinolones, 2 sulfonamides, 2 diaminopyrimidines, and 1 macrolide) in muscle tissue of farmed fish (European sea bass and gilt-head sea bream). The applied method demonstrated acceptable recovery values, mostly between 70 and 120%, with limits of quantification of the method meeting the established EU maximum residue limits. The analysis of twenty fish samples in duplicate revealed that most antibiotics were not present, with the only exception of oxolinic acid and tilmicosin in European sea bass, which were below the limit of quantification of the method.

## 1. Introduction

In response to the increasing consumer demand for fish, the decline in wild capture fisheries, and the recognized high nutritional value and numerous health benefits associated with fish consumption, aquaculture has experienced a rapid expansion, becoming one of the fastest-growing sectors in global food production [1]. In light of these developments, the Food and Agriculture Organization of the United Nations (FAO) advocated in 2018 for the incorporation of sustainable principles into the design of animal production systems [2,3]. In this regard, strong emphasis has been made regarding the responsible utilization of natural resources and the overarching goal of environmental protection [3]. This integrated framework is further enriched by considerations of food ethics [4]. Within this context, various health organizations have consistently drawn attention to the repercussions of the excessive use of veterinary drugs [5] and substances commonly employed in the treatment and prevention of infectious diseases and in growth additives [5]. This concerted effort is crucial not only for meeting the growing demand for fish but also for fostering sustainable development that aligns with environmental preservation, cultural conservation, and ethical food production practices. The delicate balance between meeting consumer needs and safeguarding the health of ecosystems, societies, and individuals requires a thoughtful and holistic approach to aquaculture and animal production management.

In any economic and technological advancement, it is imperative to conduct a thorough assessment of its impact on the ecosystem and human health. The ongoing progress in technologies and analytical methods has played a pivotal role in facilitating the swift identification and precise quantification of various substances known to be hazardous or detrimental to flora, fauna, and public health integrity [6]. In the realm of aquaculture, the predominant classes of antibiotics employed include quinolones (QNs), amphenicols, and sulfonamides (SAs), which can potentially end up in residual amounts in products consumed by the general public [7]. While the European Union Commission Regulation 37/2010 establishes maximum residue limits (MRLs) for veterinary drugs in animal-derived foodstuffs [8], including specific MRLs for fish muscle, there are still antibiotics in which MRLs still have to be established, highlighting the importance of monitoring these unregulated compounds in order to provide sufficient evidence to the authorities to apply sensible limitations. Overall, common antibiotics have MRLs that range from 30 to 600 ng/g in the case of QNs, 0.5 to 50 ng/g for diaminopyrimidines (DAPs), 100 ng/g for SAs, and 75 to 200 ng/g for macrolides (MLs) [8]. Consequently, to target these potentially harmful substances in aquatic animals, the development of multiresidue methods is imperative. These methods should exhibit high sensitivity and selectivity to accurately determine the levels of these organic contaminants in fish samples, particularly at ng/g concentrations, to ensure the safety and compliance of aquaculture products with established regulatory standards.

The contamination of fish muscle by emerging pollutants has been extensively explored in the literature, given that it constitutes the primary part of the fish consumed [9,10,11]. However, recent studies have started to focus on target analysis screening in fish liver. This shift is attributed to the role of the liver as a primary site of metabolism, leading to higher bioconcentrations of pharmaceutical compounds compared with muscle [4]. Nonetheless, the assessment of these compounds in muscle tissues is still pivotal, as regulations have strict MRLs that must be met [8]. To date, a good number of analytical methods for the determination of antibiotic residues in aquaculture products have been reported in the literature, employing diverse extraction and analysis techniques [12,13,14,15]. Concerning fish tissues, various extraction protocols have been widely utilized including solid-phase extraction, solid-phase microextraction, liquid–liquid microextraction, and solid–liquid extraction among many other approaches [12,13,14,15,16]. Among them, it is noteworthy to mention the QuEChERS (quick, easy, cheap, effective, rugged, and safe) method, which was originally developed in 2003 for the extraction of pesticides [17] but that has been subject to constant evolution throughout all these years [18], allowing its successful application in the extraction of residual quantities of antibiotics from food samples [16]. Overall, these extraction procedures commonly use solvents like acetonitrile (ACN) in combination with different types of salts (MgSO_4_, NaCl, etc.) [18], followed by clean-up steps (using different salts and sorbents such as MgSO_4_, primary secondary amine (PSA), C_18_, graphited carbon black, etc.) to remove any interferents, lipids, and proteins, etc., and to improve overall recovery of more polar compounds [16,18]. QuEChERS ensures a significant reduction in analysis time, eliminating the need for additional sample preparation and purification methods, and providing high recovery values for a broad spectrum of antibiotics, while its simplicity guarantees high reliability and reproducibility [16].

In terms of quantitative determination, antibiotics are typically studied using liquid chromatography (LC) in combination with various detectors, including ultraviolet [19,20,21], fluorescence (FLD) [22,23] and diode array detectors (DAD) [24,25,26], or tandem mass spectrometry (MS/MS) [27,28,29] to name a few. As an example, Habibi et al. [30] developed a method employing HPLC-DAD for the rapid and straightforward analysis of three macrolides in the muscle tissue of aquaculture fish (sea bream). This method has been applied to monitor products from Tunisian aquaculture. Similarly, Ghorbel-Abid et al. [31] devised a method for the simultaneous analysis of residues from five tetracyclines in the muscle tissue of farmed fish. The study reported the highest concentrations for chlortetracycline, reaching values of 137 ng/g in one sample, with the lowest content recorded for chlortetracycline at 47.4 ng/g. Analytical methods using LC-MS/MS systems are increasingly used in the determination of residues of several classes of veterinary drugs in food matrices [24,27,32,33,34] due to their high selectivity and sensitivity, allowing the simultaneous detection of positive and negative ions in a single LC cycle by combining fast polarity exchange and multiple reaction monitoring (MRM) in a short time [35]. These advancements in analytical techniques and methodologies contribute significantly to the accurate detection and quantification of antibiotic residues in aquaculture products, ensuring adherence to regulatory standards and safeguarding both public health and environmental integrity.

This study aims to validate and implement a straightforward, rapid, and efficient methodology based on the combined use of the QuEChERS method with UHPLC-MS/MS for the simultaneous determination of 10 antibiotics, including 5 QNs (marbofloxacin, enrofloxacin, difloxacin, oxolinic acid, and flumequine), 2 SAs (sulfadiazine and sulfamethoxazole), 2 DAPs (diaveridine and trimethoprim), and 1 ML (tilmicosin), in a complex matrix (muscle tissue) obtained from two species of fish (namely European sea bass and gilt-head sea bream) sourced from aquaculture farms from the island of Tenerife, Canary Islands (Spain). Additionally, the developed method will be applied to assess the presence of these compounds in a significant number of fish samples (n = 20) and determine the potential exposure risk.

## 2. Materials and Methods

### 2.1. Reagents and Materials

All reagents and solvents were LC-MS grade. ACN and methanol (MeOH) were purchased from Honeywell Inc. (Charlotte, NC, USA). Reagent-grade formic acid for LC-MS Merck KGaA (Darmstadt, Germany) and sodium acetate from VWR International Eurolab and octadecyl silicon dioxide C_18_ were from Agilent Technologies (Santa Clara, CA, USA). Anhydrous MgSO_4_ (purity 97%) was from Sigma-Aldrich (Madrid, Spain) and C_18_ (40 µm) was from Agilent Technologies (Santa Clara, CA, USA). Milli-Q gradient water was obtained from an A10 system from Millipore (Burlington, MA, USA).

Standards of difloxacin (Sigma-Aldrich; 98.6%; CAS 91296-86-5), enrofloxacin (Sigma-Aldrich; 99.8%; CAS 93106-60-6), marbofloxacin (Sigma-Aldrich; 98.5%; CAS 115550-35-1), flumequine (Sigma-Aldrich; 99.4%; CAS 42835-25-6), oxolinic acid (Sigma-Aldrich; 99.5%; CAS 14698-29-4), tilmicosin (Sigma-Aldrich; 90.2%; CAS 108050-54-0), diaveridine (Sigma-Aldrich; 99.0%; CAS 5355-16-8), trimethoprim (Sigma-Aldrich; 99.7%; CAS 738-70-5), sulfadiazine (Sigma-Aldrich; 99.7%; CAS 68-35-9), and sulfamethoxazole (Dr. Ehrenstorfer; 99.7%; CAS 723-46-6) were purchased from Sigma-Aldrich and Dr. Ehrenstorfer. Table S1 in Supplementary Materials shows the chemical structures and properties of the studied analytes.

Standard stock solutions were prepared in MeOH at a concentration of 100 mg/L and stored in amber flasks at −18 °C. Mixed working solutions were prepared through dilution with MeOH at a concentration of 1 mg/L and stored as previously mentioned. 

Volumetric glassware was cleaned by a 12 to 24 h immersion in a solution of sulfuric acid (95% *w*/*w*, VWR International, Suwanee, GA, USA) containing Nochromix (Godax Laboratories; Cabin John, MD, USA), while nonvolumetric glassware underwent a heat treatment process at 550 °C for at least 4 h using a Carbolite CWF 11/13 oven.

A Mega Star 3.0R centrifuge (VWR International Eurolab, Barcelona, Spain), a Rear Top vortex (VWR International Eurolab), and a Sartorius 224i-1S analytical balance (Goettingen, Germany) were employed across distinct phases of the extraction procedure.

### 2.2. UHPLC-MS/MS Analysis

Determination of the analytes involved chromatographic separation using an ultra-high performance liquid chromatograph (model 1260 Infinity II) fitted with a quaternary pump (model 1260 Infinity II), a temperature-controlled vial sampler (model 1260 Infinity II), a column thermostat (model 1260 MCT), and a triple quadrupole MS/MS detector (model G6470B) operating in dynamic MRM mode. This system was equipped with a jet stream electrospray ionization source (ESI), manufactured by Agilent Technologies. The mobile phase was composed of ACN and Milli-Q water, both with 0.3% (*v*/*v*) formic acid (LC-MS grade, 98–100%), and the flow rate was set at 0.5 mL/min. Separation was performed at 45 °C using an Infinity Lab Poroshell 120 EC-C_18_ column (100 × 2.1 mm, 2.7 µm) and an Infinity Lab Porerpshell EC-C_18_ guard column (5 × 2.1 mm, 2.7 µm), both from Agilent Technologies. The chromatographic separation program started with an initial mobile phase composition of water/ACN (88:12; *v*/*v*). These initial conditions were maintained for 1 min, then the organic phase was increased to 80% in a time spam of 7 min. After that, the organic phase was raised to 82.5% in 0.5 min, before returning to the initial conditions in 0.5 min and keeping the conditions for 2 min before the next injection. The total run time of the analysis was 9 min, and the injection volume was 5 µL. Regarding the ESI source parameters, the positive mode was used, with the drying gas temperature set at 300 °C with a flow rate of 7 L/min. The nebulizer pressure was 20 psi, and the sheath gas temperature was 400 °C with a flow rate of 11 L/min. The capillary voltage was set at 2500 V and the nozzle voltage at 0 V. Supplementary Materials contain Table S2, which displays the m/z values for both the quantifier and qualifier, alongside the collision energy and polarity settings utilized in the source for every analyte.

The UHPLC-MS/MS system was managed using Agilent MassHunter Workstation Data LC/MS Data Acquisition software (version 10.1, build 10.1.67). Chromatogram extraction was executed utilizing Agilent MassHunter Workstation Qualitative Analysis (version 10.0, build 10.0.10305.0), while integration and data extraction processes were performed using Agilent MassHunter Workstation Quantitative Analysis (version 10.1, build 10.1.733.0).

### 2.3. Sample Pretreatment and Storage

Two different species of fish were selected for the assessment of antibiotics in aquaculture products: European sea bass and gilt-head sea bream, both raised in fish farms on the island of Tenerife, Canary Islands (Spain). These samples were purchased by qualified academic staff from local supermarkets and placed in individual Ziploc bags labeled with a unique laboratory identifier. Upon reception at the laboratory facilities, the fish samples were thawed (when applicable), and subsequently underwent scale, head, and tail removal. Then, muscle tissues were cut with a stainless-steel knife (VWR International Eurolab), frozen with liquid nitrogen, and ground inside a metal container using a hand blender to obtain a homogeneous powder. The use of liquid nitrogen is a key factor to take into consideration in the sample pretreatment as it avoids sample heating (which may lead to the degradation of thermal labile analytes) and improves homogenization, as it makes tissues brittle [36]. After homogenization, samples were kept in individual glass containers and stored in a freezer at −18 °C until the analysis was performed.

### 2.4. QuEChERS Extraction Method

**Figure 1:**
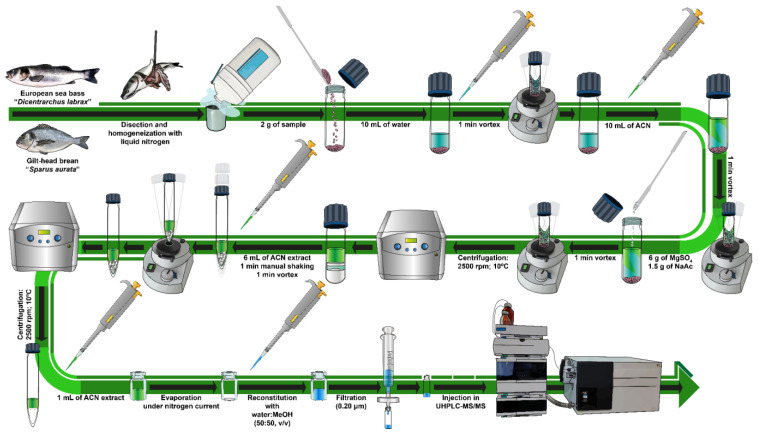
General scheme of the sample pretreatment and QuEChERS-UHPLC-MS/MS method applied in this work.

The target analytes were extracted from the fish samples using a modified QuEChERS method adapted from an Agilent Technologies application [37], focusing on the analysis of shrimp samples. Overall, the extraction procedure was as follows: after reaching room temperature, 2 g of homogenized muscle sample (wet weight, ww) were weighed and placed in a 50 mL glass centrifuge tube. Then, the sample was spiked with the target analytes by adding the pollutants solution on top of the sample (ensuring that the surface of the sample was directly in contact with it) and left for 30 min before the application of the QuEChERS method. After that, 10 mL of water were added and the mixture was shaken in a vortex for 1 min. Afterwards, 10 mL of ACN (5% formic acid) were incorporated, and the tube was vortexed again for another minute. Subsequently, 6 g of anhydrous MgSO_4_ and 1.5 g of anhydrous sodium acetate were added to the mixture, and it was vigorously vortexed for 1 min and centrifuged at 2500 rpm for 10 min at 10 °C. After this, for the clean-up step, 6 mL of the ACN extract were transferred into a 15 mL centrifuge tube containing 150 mg of C_18_ and 900 mg of anhydrous MgSO_4_. The tube was vortexed for 1 min and then centrifuged (2500 rpm, 10 min; 10 °C). Next, 1 mL of the supernatant solution was transferred into a 10 mL clear glass tube and evaporated to dryness under a nitrogen current. The residue was reconstituted in 1 mL MeOH/water (50:50, *v*/*v*) and filtered through a 0.20 µm polyvinylidene fluoride (PVDF) filter before injecting in the UHPLC-MS/MS system. Figure 1 shows the general scheme of the sample pretreatment and the extraction procedure applied in this work.

## 3. Results and Discussion

### 3.1. UHPLC-MS/MS Figures of Merit

As already mentioned in the experimental section, the separation and detection of the 10 target analytes was achieved through a UHPLC-MS/MS system. Chromatographic separation and MS detection conditions were optimized with standard solutions of QNs, DAPs, SAs, and the macrolide, using the gradient program previously described, and using a C_18_ column (100 × 2.1 mm, 2.7 µm) from Agilent Technologies, also previously mentioned. Focusing on previous experience from our research groups and bibliographic data, ACN and water were selected as the components of the mobile phase, both containing a small amount of formic acid (0.3%) [38,39].

To ensure the precision of the injection in the UHPLC-MS/MS system, intra- and inter-day repeatability studies were carried out at three concentration levels (25, 40, and 80 μg/L). In this sense, the three antibiotic mixtures were injected in quintuplicate (n = 5) for three consecutive days (n = 15). The results displayed in Tables S3 and S4 in Supplementary Materials show that the injections in the UHPLC-MS/MS system are highly repeatable, displaying relative standard deviation (RSD) values lower than 0.2% for retention times and 4.1% for peak areas on the intra-day studies, and less than 0.32% and 15.1% for the same parameters on the inter-day studies.

To evaluate the linearity of the method, a calibration curve in the reconstitution mixture (MeOH/water; 50:50, *v*/*v*), comprising 10 concentration levels, was carried out. The instrumental calibration data for the target analytes can be found in Table S5 in Supplementary Materials. Overall, the lowest calibration levels (LCLs) were used due to the favorable signal-to-noise (S/N) ratios achieved in the UHPLC-MS/MS system, ensuring a S/N ratio exceeding 10. For the instrumental calibration of the different analytes, the established LCLs were between 3 and 10 µg/L, while the studied linear range spanned up to 80 to 100 µg/L. In all cases, the determination coefficients (R^2^) for the analytes exceeded 0.990. In Figure S1 in Supplementary Materials, the MRM chromatogram shows the chromatographic separation of various analytes at 80 µg/L in the reconstitution mixture (MeOH/water; 50:50, *v*/*v*), displaying well-defined and symmetric peaks. The total analysis time for all the analytes was less than 9 min.

### 3.2. Recovery Studies in Fish Samples

Using the QuEChERS extraction conditions previously mentioned, a complete evaluation of the performance of the method was conducted, assessing the recovery of the analytes in both fish species (European sea bass and gilt-head sea bream) at three concentration levels (125, 200, and 400 ng/g; final concentrations of 25, 40, and 80 µg/L). Absolute recovery values were calculated by determining the ratio between the areas of the extracted spiked samples and those of the matrix-matched standard at the final theoretical concentration. Table 1 shows the recovery values obtained for each sample at the different concentration levels for the different analytes assessed in this work. Overall, good recovery values were obtained for most of the analytes. Absolute average recovery values ranged between 63.0 and 97.4% with RSD values below 21%, except for sulfadiazine in gilt-head sea bream and marbofloxacin in both types of matrices, which were slightly lower (58.3% in the first case and 44.7% and 51.6% in the second). Based on the results obtained, it can be affirmed that the proposed method exhibits a comparable performance in both fish species, revealing high recovery rates for most analytes, accompanied by low RSD values, indicating its suitability for the analysis of antibiotics in fish. These results, with some exceptions, align with typical criteria employed in the development of extraction methods. As indicated in the SANTE guidelines [40], ideally, recovery values should be within a 70–120% range with RSDs ≤ 20%; however, there are specific situations when a more extensive range may be deemed acceptable (30–140%). Although initially developed for pesticides, the criteria outlined in the SANTE guidelines offer a robust framework that can be effectively applied to guide the development of diverse analytical methodologies.

### 3.3. Matrix-Matched Calibration and Matrix Effect Evaluation

Subsequently, a matrix-matched calibration was developed for all analytes in both matrices (European sea bass, Figure S2 in Supplementary Materials; and gilt-head sea bream, Figure S3 in Supplementary Materials) at ten increasing concentration levels (n = 10), injecting five times (n = 5) each level. Analytes were studied in a linear range starting from 3–10 µg/L up to 80–100 µg/L. Overall, higher LCLs were observed for diaveridine, trimethoprim, and oxolinic acid (5 µg/L) and enrofloxacin and difloxacin (10 µg/L). Due to the favorable absolute recovery values achieved for most analytes, the method yielded low limits of quantification (LOQs) ranging from 20.5 to 68.6 ng/g, except for marbofloxacin, whose LOQs were between 96.9 and 111.9 ng/g in gilt-head sea bream and European sea bass, respectively. These LOQs were calculated based on the LCLs of the matrix-matched calibration curves, the dilution factor, and considering the absolute recovery values for each analyte in both matrices. Experimental verification of these values was performed through extractions at comparable concentrations. Detailed results for the LOQs of the method are presented in Table S6 in Supplementary Materials.

**Figure 2:**
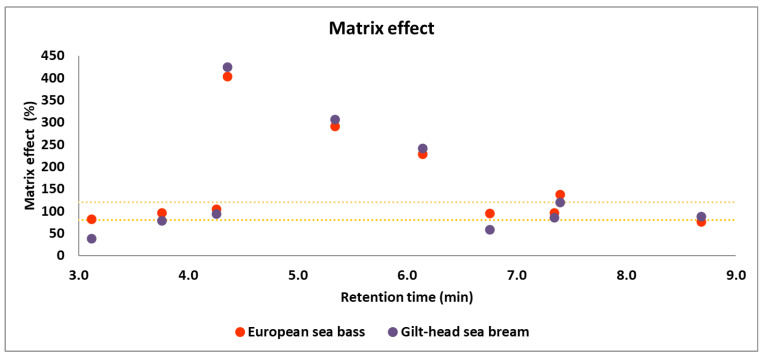
Distribution of the matrix effect (%) vs. the retention time (minutes) of each antibiotic in European sea bass and gilt-head sea bream matrices after the application of the QuEChERS-UHPLC–MS/MS method. The yellow dot lines indicate matrix effects values of 80 and 120%.

Given the complexity of the samples studied, as well as the possible differences among matrices, an assessment of the matrix effect was conducted for both fish species. The evaluation employed the equation proposed by Matuszewski et al. [41]. Specifically, the matrix effect was determined by calculating the average ratio between the area of the analyte in the matrix-matched calibration and its area in the solvent calibration at each concentration level of the calibration curves. According to Matuszewki’s approach, a matrix effect is considered negligible when values fall within a range from 80 to 120%. Values exceeding this range indicate a substantial signal enhancement, while values below it indicate significant signal suppression. It is imperative to consider this effect in both matrices to ensure accurate interpretations of the analytical results. As depicted in Figure 2, some analytes exhibit noteworthy signal enhancement (difloxacin, enrofloxacin, and marbofloxacin in both matrices and tilmicosin in European sea bass), while others show substantial signal suppression (sulfadiazine, diaveridine, and sulfamethoxazole in gilt-head sea bream and flumequine in European sea bass), therefore matrix-matched calibration should be developed for quantification purposes. Overall, the matrix effect on both types of samples was quite similar; however, it was decided to carry out independent matrix-matched calibrations for quantitation purposes due to the differences observed. The significant matrix effect observed for many of the analytes highlights the importance of developing matrix-matched calibrations for the determination of the antibiotics in real samples.

### 3.4. Analysis of Real Samples

To assess the impact of various antibiotics in farmed fish samples, 20 samples were analyzed (10 from each species of fish) in duplicate. Due to the significant matrix effect observed for several analytes (refer to Figure 2), quantification was performed using matrix-matched calibration. After a thorough assessment, the results indicated that most of the analytes were not detected (see Table S7 in Supplementary Materials), with the only exception of tilmicosin (a macrolide antibiotic that can be used in aquaculture for treating bacterial kidney disease [42]), which was detected in two European sea bass samples, and oxolinic acid (an extensively used quinolone antibiotic employed in aquaculture to combat bacterial infections in fish [43]), which was detected in all European sea bass samples (see Figure S4 in Supplementary Materials). Nevertheless, the concentrations found were lower than the LOQ of the method, hence below current MRLs established by European legislation (50–100 ng/g) [8,44,45]. The detection of these compounds, albeit below regulatory limits, underscores the importance of ongoing monitoring efforts to ensure environmental and food safety. Concerning the assessment of environmental impact, numerous countries worldwide tightly control and regulate the usage of pharmaceutical drugs, with guidelines often enforced by health agencies or veterinary professionals. Despite these regulations, a significant portion of global aquaculture production (90%) in certain countries lacks adequate control measures. Furthermore, continuous and extensive data on the usage of antibiotics in aquaculture will always be required to ensure high quality food that meets the required safety standards enforced in different nations around the world [46].

## 4. Conclusions

The QuEChERS extraction method was successfully applied for the simultaneous determination of 10 antibiotics in muscle samples from two fish species (European sea bass, and gilt-head sea bream). The validated QuEChERS-LC-MS/MS method provided a simple and efficient solution for the determination of antibiotics. It showed good recovery values for most of the antibiotics with low RSDs and LOQs of the method that met the required MRLs established by the European Union for the target analytes. The method was applied to the analysis of twenty fish samples sourced from local aquaculture farms in the island of Tenerife, Canary Islands (Spain). Overall, results did not show the presence of any of the studied analytes. The only exceptions were tilmicosin and oxolinic acid in European sea bass, which could be identified in several fish muscles samples but at concentrations below the LOQ, lower than current MRLs. Finally, although the current method performed well in terms of recovery and precision in most of the antibiotics, it may be further tested, and consequently adapted, for the extraction of other veterinary dugs used in aquaculture farms. Additionally, it could be used to analyze other emerging organic pollutants, including pharmaceuticals, personal care products, phthalic acid esters, and other types of compounds in order to obtain an extensive vision of the pollutants that may be affecting fish in aquaculture farms.

## Figures and Tables

**Table 1:** Absolute recovery and RSD values of the target analytes in European sea bass and gilt-head sea bream (n = 5 at each spiking level).

Analytes	Sample	125 ng/g	200 ng/g	400 ng/g	Mean
		Recovery % (RSD %)	Recovery % (RSD %)	Recovery % (RSD %)	Recovery % (RSD %)
Sulfadiazine	European sea bass	77.5 (8.5)	**52.3** (13)	76.5 (1.6)	68.8 (**21**)
	Gilt-head sea bream	**52.3** (18)	**55.6** (1.9)	66.9 (17)	**58.3** (13.1)
Diaveridine	European sea bass	90.6 (3.6)	95.4 (1.6)	94.3 (0.4)	93.4 (2.7)
	Gilt-head sea bream	90.9 (6.8)	99.9 (2.0)	92.9 (8.1)	94.6 (5.0)
Trimethoprim	European sea bass	87.0 (4.1)	95.5 (0.6)	94.7 (0.2)	92.4 (5.1)
	Gilt-head sea bream	91.7 (4.4)	98.5 (1.2)	93.0 (5.9)	94.4 (3.8)
Marbofloxacin	European sea bass	**41.5** (5.5)	**43.8** (12)	**48.7** (5.4)	**44.7** (8.2)
	Gilt-head sea bream	**40.1** (10)	**49.9** (5.4)	64.7 (16)	**51.6** (**24**)
Enrofloxacin	European sea bass	67.4 (11)	74.1 (12)	77.3 (2.8)	72.9 (6.9)
	Gilt-head sea bream	73.8 (4.5)	82.8 (6.1)	83.1 (19)	79.9 (6.6)
Difloxacin	European sea bass	80.9 (6.1)	81.1 (10)	83.9 (2.7)	81.9 (2.0)
	Gilt-head sea bream	81.2 (3.3)	87.1 (5.6)	86.8 (15)	85.0 (3.9)
Sulfamethoxazole	European sea bass	85.7 (8.9)	**55.7** (9.6)	77.6 (2.2)	73.0 (**21**)
	Gilt-head sea bream	**55.6** (14)	61.6 (4.6)	71.7 (13)	63.0 (13)
Oxolinic acid	European sea bass	82.4 (6.1)	80.9 (19)	83.6 (2.4)	82.3 (1.7)
	Gilt-head sea bream	80.5 (5.2)	85.4 (10)	81.5 (4.4)	82.5 (3.1)
Tilmicosin	European sea bass	71.1 (17)	95.8 (2.0)	93.0 (1.4)	86.6 (16)
	Gilt-head sea bream	93.4 (4.0)	98.4 (1.5)	100 (6.4)	97.4 (3.7)
Flumequine	European sea bass	82.0 (4.4)	80.3 (18)	88.9 (2.4)	83.7 (5.4)
	Gilt-head sea bream	86.9 (3.8)	87.1 (9.7)	103 (**21**)	92.1 (10)

In bold, recovery values below 60% and RSD values above 20%.

## Data Availability

The original contributions presented in the study are included in the article and Supplementary Material, further inquiries can be directed to the corresponding authors.

## Supplementary Materials

The following supporting information can be downloaded at: https://www.mdpi.com/article/10.3390/foods13071081/s1, Figure S1. UHPLC-MS/MS dynamic MRM chromatogram at 400 ng/g of the different analytes studied in this paper. Figure S2. Calibration curves of the different analytes in European seabass. Figure S3. Calibration curves of the different analytes in gilt-head seabream. Figure S4. Representative quantifier and qualifier UHPLC-MS/MS dynamic MRM chromatograms of two of the analytes detected (below the LOQ of the method) in real samples. Quantifier peaks, delineated by a black contour line, and qualifier peaks, delineated by a blue contour line. All samples exhibited a S/N ratio equal or higher than 10 and quantifier/qualifier ratios were satisfactory. Table S1. Chemical structure and properties of the studied antibiotics. Table S2. Operational MS/MS conditions and the m/z transitions of the target compounds. Table S3. Results of the UHPLC-MS/MS intra-day and inter-day precision study for the peak areas. TableS4. Results of UHPLC-MS/MS intra-day and inter-day precision study for the retention times. Table S5. Solvent and matrix-matched calibration data of the target analytes. Table S6. Limits of quantification of the method in European sea bass and gilt-head sea bream. Table S7. Concentrations of the different target analytes found in European sea bass (n = 10) and gilt-head sea bream (n = 10) samples.
