# Supplementary material for: Determination of Multiclass Antibiotics in Fish Muscle Using a QuEChERS-UHPLC-MS/MS Method
Source: Foods. 2024 Apr 1;13(7):1081. doi: 10.3390/foods13071081 (PMC11012132; doi:10.3390/foods13071081)
Supplement: Supplementary file 1 [file foods-13-01081-s001.zip › foods-2909841-supplementary.pdf]

Determination of Multiclass Antibiotics in Fish Muscle Using a QuEChERS-UHPLC-MS/MS Method

Yousra Aissaoui [1,2], Gabriel Jiménez-Skrzypek [2,3], Javier González-Sálamo [2,3], Malika Trabelsi-Ayadi [4], Ibtissem Ghorbel-Abid [1,4,*] and Javier Hernández-Borges [2,3,*]

[1] Useful Materials Laboratory (LMU), National Institute for Physical and Chemical Research and Analysis (INRAP), Ariana 2020, Tunisia; yousra.issaoui@fsb.ucar.tn
[2] Departamento de Química, Unidad Departamental de Química Analítica, Facultad de Ciencias, Universidad de La Laguna (ULL), Avda. Astrofísico Fco. Sánchez, s/n, 38206 San Cristóbal de La Laguna, Spain; gjimenez@ull.edu.es (G.J.-S.); jgsalamo@ull.edu.es (J.G.-S.)
[3] Instituto Universitario de Enfermedades Tropicales y Salud Pública de Canarias, Universidad de La Laguna (ULL), Avda. Astrofísico Fco. Sánchez, s/n, 38206 San Cristóbal de La Laguna, Spain
[4] Laboratory of Application Chemistry to the Resources and Natural Substances and the Environment (LACReSNE), Faculty of Science of Bizerte, University of Carthage, Zarzouna, Bizerte 7021, Tunisia; malika.trabelsiayadi@gmail.com
* Correspondence: ibtissem.gh.ab@gmail.com (I.G.-A.); jhborges@ull.edu.es (J.H.-B.)

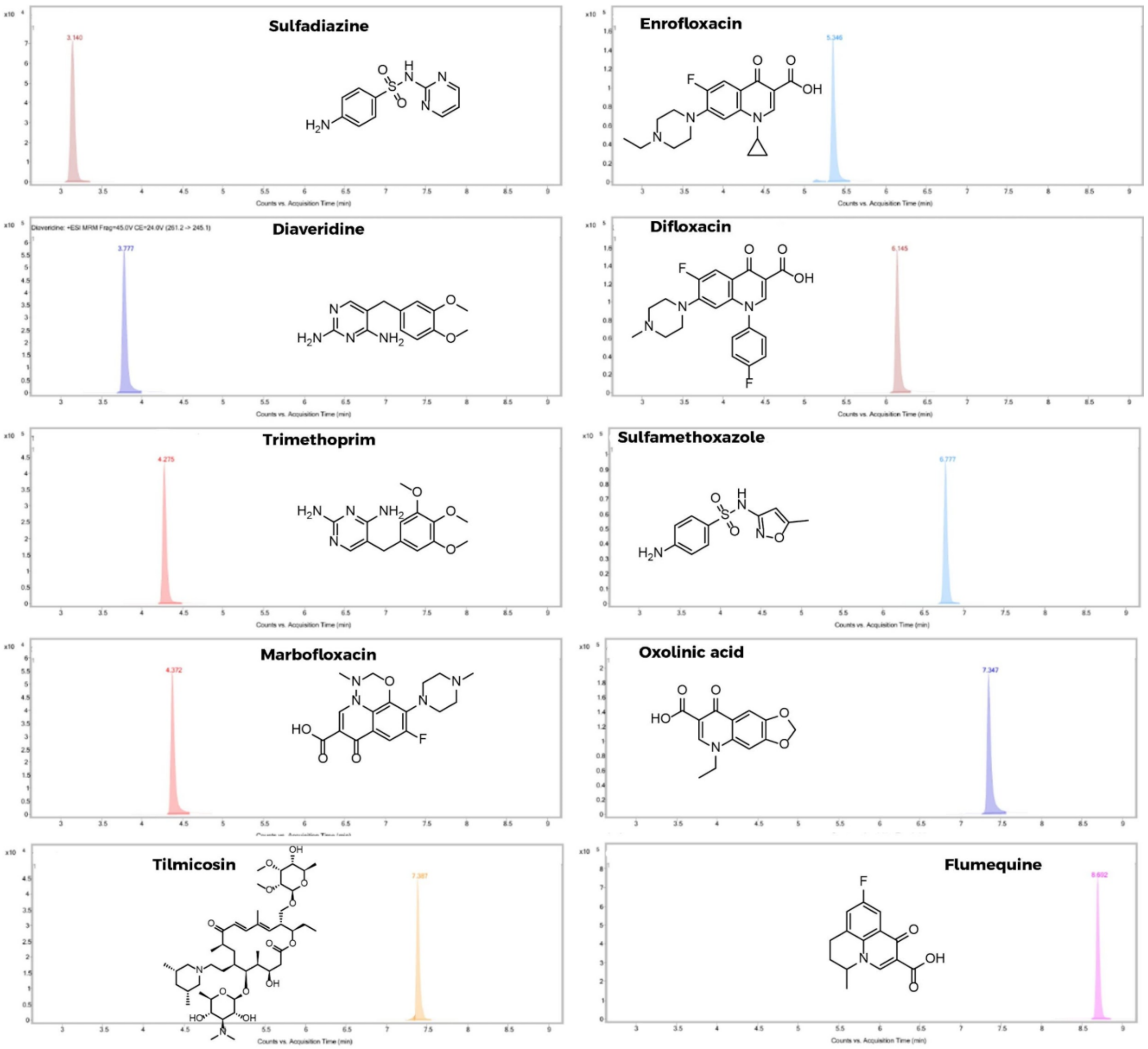

Figure S1. UHPLC-MS/MS dynamic MRM chromatogram at 400 ng/g of the different analytes studied in this paper.

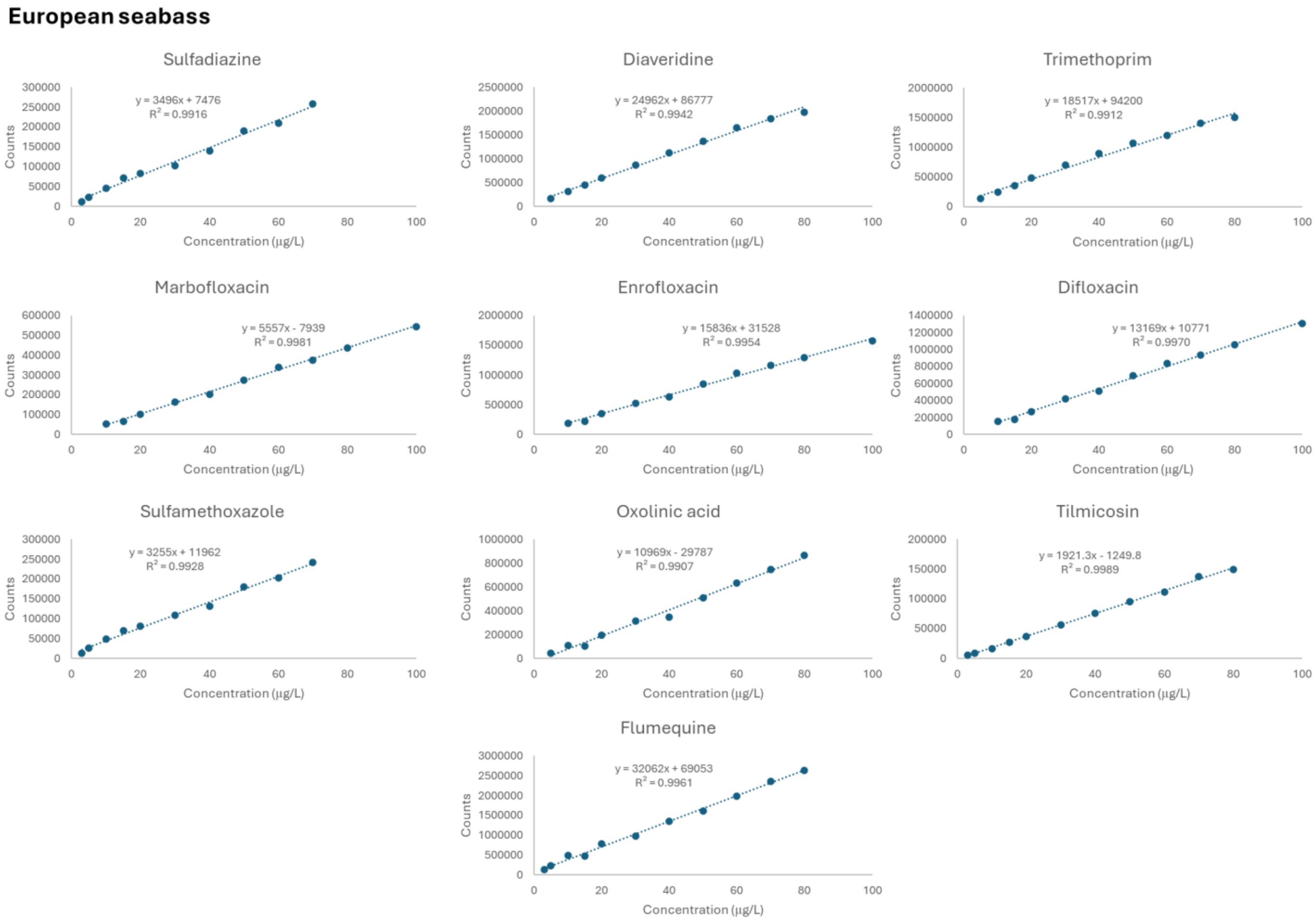

Figure S2. Calibration curves of the different analytes in European seabass.

Gilt-head bream

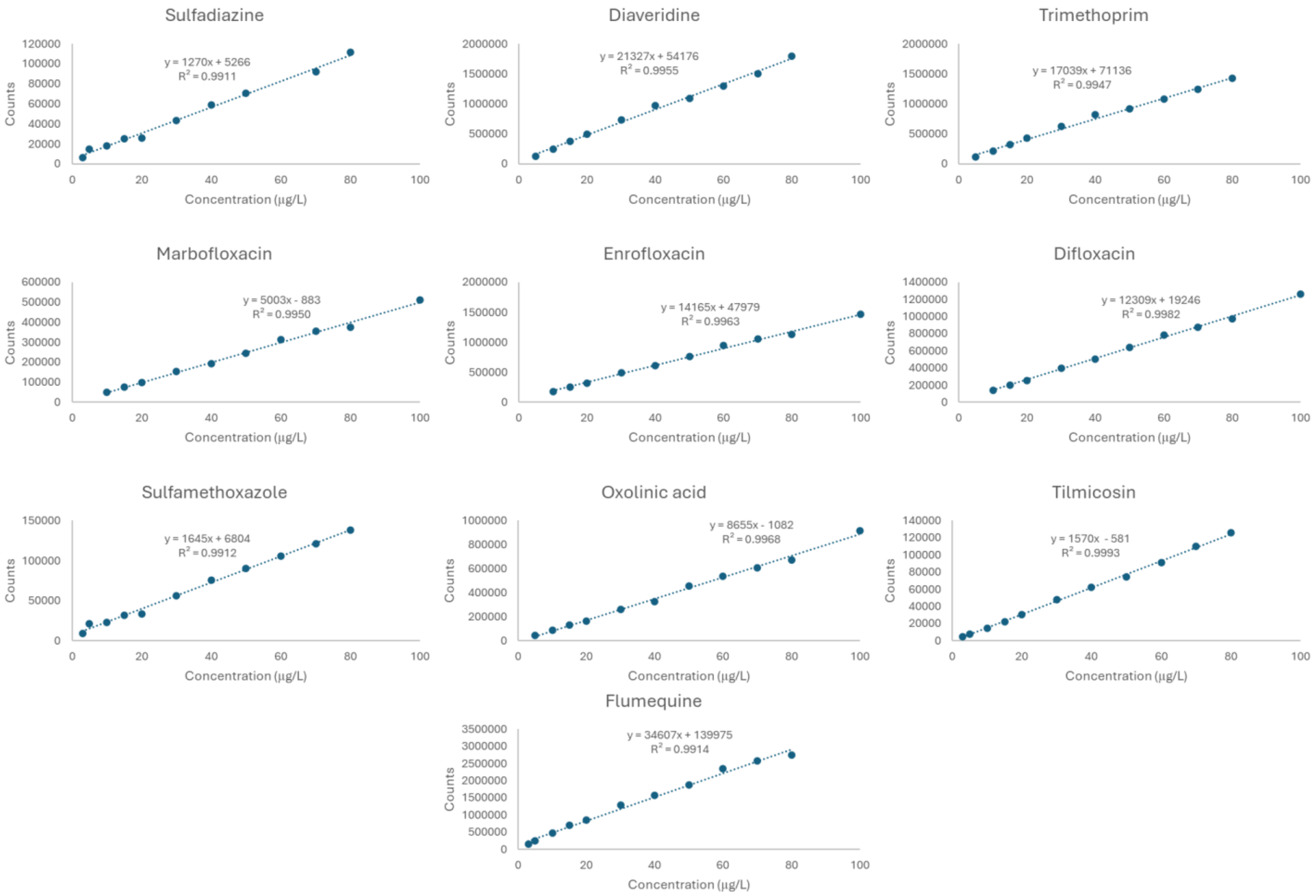

Figure S3. Calibration curves of the different analytes in gilt-head seabream.

European Seabass

Oxolinic acid

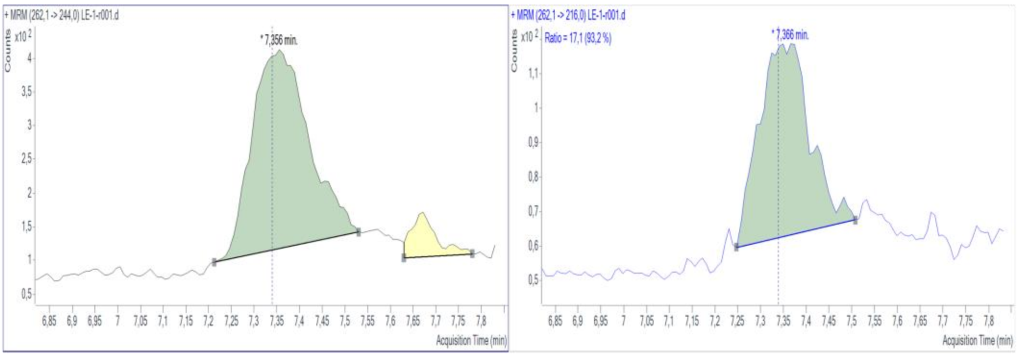

Sample 5

Tilmicosin

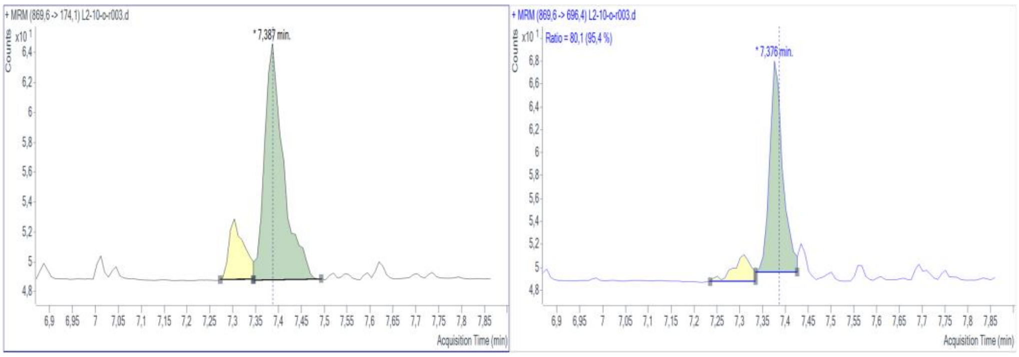

Sample 9

Figure S4. Representative quantifier and qualifier UHPLC-MS/MS dynamic MRM chromatograms of two of the analytes detected (below the LOQ of the method) in real samples. Quantifier peaks, delineated by a black contour line, and qualifier peaks, delineated by a blue contour line. All samples exhibited a S/N ratio equal or higher than 10 and quantifier/qualifier ratios were satisfactory.

Table S1.- Chemical structure and properties of the studied antibiotics.

Analyte	Structure	Molecular formula	MM (g/mol)	Solubility in water (25 ºC)	Vapor pressure (mmHg, 25 ºC)	Log K_{OW}	Melting point (ºC)	Boiling point (ºC)
Sulfadiazine	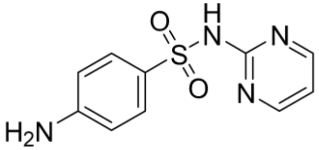	$C_{10}H_{10}N_4O_2S$	250.28	77 mg/L	$1.28·10^{-10}$	-0.09	255.5	512.6
Diaveridine	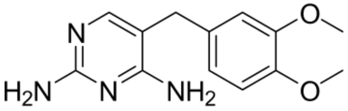	$C_{13}H_{16}N_4O_2$	260.29	-	$2.29·10^{-10}$	0.97	233	506.1
Trimethoprim	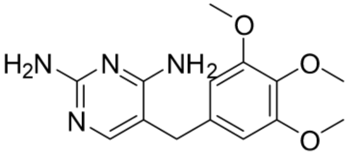	$C_{14}H_{18}N_4O_3$	290.32	<1 g/L	0.0 ± 0.9	0.91	199-203	526
Marbofloxacin	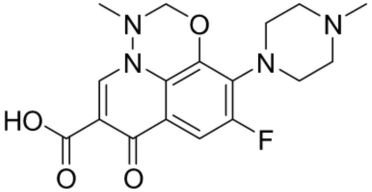	$C_{17}H_{19}FN_4O_4$	362.36	-	$7.45·10^{-10}$	-0.5	268-269	571
Enrofloxacin	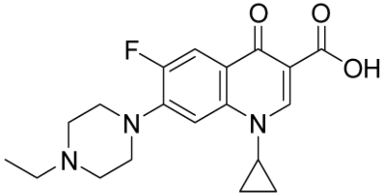	$C_{19}H_{22}F_1N_3O_3$	359.40	> 53.9 mg/L	-	0.70	221-226	560.5

Analyte	Structure	Molecular formula	MM (g/mol)	Solubility in water (25 ºC)	Vapor pressure (mmHg, 25 ºC)	Log Kow	Melting point (ºC)	Boiling point (ºC)
Difloxacin	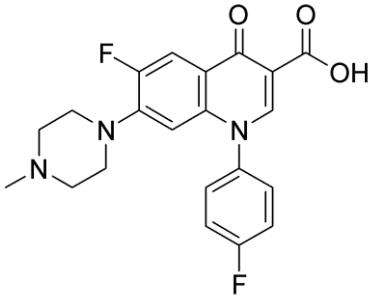	$C_{19}H_{20}FN_3O_3$	357.38	-	-	0.89	322.44	595.5
Sulfamethoxazole	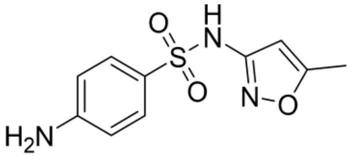	$C_{10}H_{11}N_3O_3S$	253.28	<1 g/L	-	0.89	166-169	482.1
Oxolinic acid	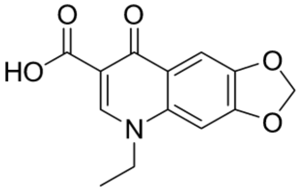	$C_{13}H_{11}NO_5$	261.23	-	-	0.94	314-316	473.2

Analyte	Structure	Molecular formula	MM (g/mol)	Solubility in water (25 ºC)	Vapor pressure (mmHg, 25 ºC)	Log K_{OW}	Melting point (ºC)	Boiling point (ºC)
Tilmicosin	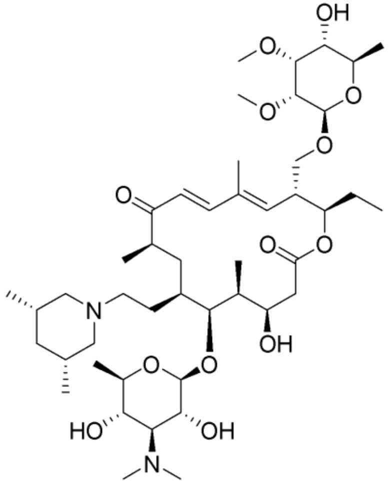	$C_{46}H_{80}N_2O_{13}$	869.13	-	-	3.8	-	926.6
Flumequine	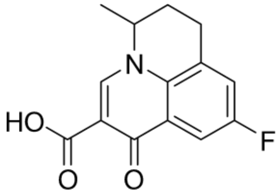	$C_{14}H_{12}FNO_3$	252	-	-	1.6	253-255	439.7

Data taken from SciFinder® and PubChem databases. MM: Molecular mass.

"-", data not available.

Table S2. Operational MS/MS conditions and the m/z transitions of the target compounds.

Analyte		Precursor ion (m/z)	Product ion (m/z)	Collision energy (eV)	Polarity
Sulfadiazine	Quantifier	251.0	156.0	10	Positive
	Qualifier	251.0	108.0	22	Positive
Diaveridine	Quantifier	261.2	245.1	24	Positive
	Qualifier	261.2	123.1	26	Positive
Trimethoprim	Quantifier	291.1	230.1	20	Positive
	Qualifier	291.1	123.1	40	Positive
Marbofloxacin	Quantifier	363.0	345.1	17	Positive
	Qualifier	363.0	320.1	9	Positive
Enrofloxacin	Quantifier	360.0	342.1	18	Positive
	Qualifier	360.0	316.2	18	Positive
Difloxacin	Quantifier	400.1	382.1	20	Positive
	Qualifier	400.1	356.2	16	Positive
Sulfamethoxazole	Quantifier	254.1	156.0	10	Positive
	Qualifier	254.1	92.0	26	Positive
Oxolinic acid	Quantifier	262.1	244.0	13	Positive
	Qualifier	262.1	216.0	30	Positive
Tilmicosin	Quantifier	869.6	174.1	40	Positive
	Qualifier	869.6	696.4	44	Positive
Flumequine	Quantifier	291.1	244.0	13	Positive
	Qualifier	291.1	202.0	33	Positive

Table S3. Results of the UHPLC-MS/MS intra-day and inter-day precision study for the peak areas.

Analyte	Level 1: 25 µg/L				Level 2: 40 µg/L				Level 3: 80 µg/L			
	Intra-day RSD (%, n=5)			Inter-day RSD (%, n=15)	Intra-day RSD (%, n=5)			Inter-day RSD (%, n=15)	Intra-day RSD (%, n=5)			Inter-day RSD (%, n=15)
	Day 1	Day 2	Day 3		Day 1	Day 2	Day 3		Day 1	Day 2	Day 3	
Sulfadiazine	0.95	0.16	0.59	14.49	0.21	0.74	0.42	13.00	1.53	0.84	0.27	12.38
Diaveridine	1.10	1.12	0.40	15.08	0.83	0.06	0.27	5.08	0.13	0.74	0.17	2.24
Trimethoprim	1.10	1.01	0.36	11.89	0.37	0.21	0.41	1.95	0.28	0.52	0.27	0.82
Marbofloxacin	1.05	1.74	1.47	10.41	0.88	2.19	1.59	12.43	2.23	1.67	1.24	8.74
Enrofloxacin	1.04	1.90	1.82	11.73	0.50	1.75	0.99	10.21	1.18	2.58	0.98	13.47
Difloxacin	1.00	0.22	0.79	13.05	0.38	1.18	0.23	10.44	0.59	1.99	0.50	14.53
Sulfamethoxazole	0.96	0.29	0.36	12.69	0.06	0.25	0.30	5.12	0.29	0.43	0.04	4.99
Oxolinic acid	2.67	4.64	1.74	3.21	1.64	4.00	1.43	2.36	0.90	2.64	0.42	13.49
Tilmicosin	0.33	1.68	0.50	13.91	0.14	1.71	0.17	5.36	0.98	1.13	0.54	2.45
Flumequine	1.69	3.04	1.07	12.35	0.27	2.13	0.55	14.07	0.68	1.89	0.20	1.15

TableS4. Results of UHPLC-MS/MS intra-day and inter-day precision study for the retention times.

Analyte	Level 1: 25 µg/L				Level 2: 40 µg/L				Level 3: 80 µg/L			
	Intra-day RSD (%, n=5)			Inter-day RSD (%, n=15)	Intra-day RSD (%, n=5)			Inter-day RSD (%, n=15)	Intra-day RSD (%, n=5)			Inter-day RSD (%, n=15)
	Day 1	Day 2	Day 3		Day 1	Day 2	Day 3		Day 1	Day 2	Day 3	
Sulfadiazine	0.15	0.00	0.00	0.14	0.00	0.15	0.00	0.14	0.00	0.15	0.00	0.19
Diaveridine	0.13	0.00	0.13	0.07	0.00	0.13	0.00	0.11	0.13	0.13	0.00	0.20
Trimethoprim	0.00	0.00	0.11	0.10	0.00	0.11	0.00	0.10	0.00	0.11	0.11	0.07
Marbofloxacin	0.11	0.00	0.11	0.04	0.00	0.11	0.00	0.16	0.11	0.00	0.00	0.16
Enrofloxacin	0.00	0.00	0.09	0.08	0.09	0.00	0.00	0.12	0.00	0.00	0.00	0.09
Difloxacin	0.00	0.08	0.08	0.05	0.00	0.08	0.00	0.15	0.08	0.00	0.00	0.11
Sulfamethoxazole	0.07	0.07	0.07	0.07	0.07	0.00	0.00	0.12	0.00	0.00	0.00	0.07
Oxolinic acid	0.07	0.00	0.07	0.04	0.07	0.00	0.00	0.09	0.00	0.00	0.00	0.07
Tilmicosin	0.07	0.00	0.07	0.37	0.07	0.06	0.00	0.31	0.00	0.00	0.07	0.18
Flumequine	0.00	0.00	0.00	0.00	0.00	0.00	0.00	0.06	0.00	0.00	0.00	0.06

Table S5.- Solvent and matrix-matched calibration data of the target analytes.

Analyte	Solvent/Matrix matched calibration	Studied linear range (μg/L)	Regression equation (n=10)		$s_{y/x}$	R^2	LCL (μg/L)
			$b \pm s_b{\cdot}t_{(0.05;8)}$	$a \pm s_a{\cdot}t_{(0.05;8)}$			
Sulfadiazine	Solvent	3-80	$4.990{\cdot}10^3 \pm 0.097{\cdot}10^3$	$-2.709{\cdot}10^3 \pm 3.657{\cdot}10^3$	2993	0.9994	3
	European sea bass	3-80	$3.496{\cdot}10^3 \pm 0.262{\cdot}10^3$	$7.476{\cdot}10^3 \pm 9.881{\cdot}10^3$	8086	0.9916	3
	Gilt-head sea bream	3-80	$1.270{\cdot}10^3 \pm 0.120{\cdot}10^3$	$5.266{\cdot}10^3 \pm 4.192{\cdot}10^3$	3136	0.9911	3
Diaveridine	Solvent	5-80	$26.017{\cdot}10^3 \pm 1.426{\cdot}10^3$	$91.995{\cdot}10^3 \pm 64.784{\cdot}10^3$	48718	0.9955	5
	European sea bass	5-80	$24.962{\cdot}10^3 \pm 1.551{\cdot}10^3$	$86.777{\cdot}10^3 \pm 70.487{\cdot}10^3$	53007	0.9942	5
	Gilt-head sea bream	5-80	$21.327{\cdot}10^3 \pm 1.165{\cdot}10^3$	$54.176{\cdot}10^3 \pm 52.960{\cdot}10^3$	39827	0.9955	5
Trimethoprim	Solvent	5-80	$17.780{\cdot}10^3 \pm 1.260{\cdot}10^3$	$86.990{\cdot}10^3 \pm 57.252{\cdot}10^3$	43054	0.9925	5
	European sea bass	5-80	$18.517{\cdot}10^3 \pm 1.423{\cdot}10^3$	$94.200{\cdot}10^3 \pm 64.671{\cdot}10^3$	48633	0.9912	5
	Gilt-head sea bream	5-80	$17.039{\cdot}10^3 \pm 1.016{\cdot}10^3$	$71.136{\cdot}10^3 \pm 46.190{\cdot}10^3$	34736	0.9947	5
Marbofloxacin	Solvent	10-100	$3.147{\cdot}10^3 \pm 0.294{\cdot}10^3$	$-36.995{\cdot}10^3 \pm 16.161{\cdot}10^3$	10396	0.9913	10
	European sea bass	10-100	$5.557{\cdot}10^3 \pm 0.197{\cdot}10^3$	$-7.939{\cdot}10^3 \pm 10.889{\cdot}10^3$	7662	0.9981	10
	Gilt-head sea bream	10-100	$5.003{\cdot}10^3 \pm 2.879{\cdot}10^3$	$-0.883{\cdot}10^3 \pm 15.931{\cdot}10^3$	11210	0.9950	10
Enrofloxacin	Solvent	10-100	$10.786\ 10^3 \pm 0.771{\cdot}10^3$	$-118.766{\cdot}10^3 \pm 42.684{\cdot}10^3$	30033	0.9924	10
	European sea bass	10-100	$15.836{\cdot}10^3 \pm 0.880{\cdot}10^3$	$31.528{\cdot}10^3 \pm 48.714{\cdot}10^3$	34276	0.9954	10
	Gilt-head sea bream	10-100	$14.165{\cdot}10^3 \pm 0.703{\cdot}10^3$	$47.979{\cdot}10^3 \pm 38.909{\cdot}10^3$	27377	0.9963	10
Difloxacin	Solvent	10-100	$10.089{\cdot}10^3 \pm 0.755{\cdot}10^3$	$-100.419{\cdot}10^3 \pm 41.779{\cdot}10^3$	29396	0.9916	10
	European sea bass	10-100	$13.169{\cdot}10^3 \pm 0.593{\cdot}10^3$	$10.771\ E{\cdot}10^3 \pm 32.834{\cdot}10^3$	23103	0.9970	10
	Gilt-head sea bream	10-100	$12.309{\cdot}10^3 \pm 0.430{\cdot}10^3$	$19.246{\cdot}10^3 \pm 23.816{\cdot}10^3$	16757	0.9982	10
	Solvent	3-80	$4.329{\cdot}10^3 \pm 0.073{\cdot}10^3$	$-2.159{\cdot}10^3 \pm 2.762{\cdot}10^3$	2260	0.9996	3

Analyte	Solvent/Matrix matched calibration	Studied linear range (μg/L)	Regression equation (n=10)		$s_{y/x}$	R^2	LCL (μg/L)
			$b \pm s_b \cdot t_{(0.05;8)}$	$a \pm s_a \cdot t_{(0.05;8)}$			
Sulfamethoxazole	European sea bass	3-80	$3.255 \cdot 10^3 \pm 0.248 \cdot 10^3$	$11.962 \cdot 10^3 \pm 9.543 \cdot 10^3$	7464	0.9928	3
	Gilt-head sea bream	3-80	$1.645 \cdot 10^3 \pm 0.155 \cdot 10^3$	$6.804 \cdot 10^3 \pm 5.407 \cdot 10^3$	4045	0.9912	3
Oxolinic acid	Solvent	5-80	$10.640 \cdot 10^3 \pm 0.282 \cdot 10^3$	$-10.192 \cdot 10^3 \pm 12.818 \cdot 10^3$	9640	0.9989	5
	European sea bass	5-80	$10.969 \cdot 10^3 \pm 0.866 \cdot 10^3$	$-29.787 \cdot 10^3 \pm 39.332 \cdot 10^3$	29578	0.9907	5
	Gilt-head sea bream	5-80	$8.655 \cdot 10^3 \pm 0.401 \cdot 10^3$	$-1.082 \cdot 10^3 \pm 18.223 \cdot 10^3$	13704	0.9968	5
Tilmicosin	Solvent	3-80	$1.487 \cdot 10^3 \pm 0.049 \cdot 10^3$	$-2.881 \cdot 10^3 \pm 2.105 \cdot 10^3$	1801	0.9983	3
	European sea bass	3-80	$1.895 \cdot 10^3 \pm 0.029 \cdot 10^3$	$-0.836 \cdot 10^3 \pm 1.139 \cdot 10^3$	960	0.9996	3
	Gilt-head sea bream	3-80	$1.570 \cdot 10^3 \pm 0.034 \cdot 10^3$	$-0.581 \cdot 10^3 \pm 1.464 \cdot 10^3$	1253	0.9993	3
Flumequine	Solvent	3-80	$39.087 \cdot 10^3 \pm 2.517 \cdot 10^3$	$164.818 \cdot 10^3 \pm 107.254 \cdot 10^3$	91774	0.9938	3
	European sea bass	3-80	$32.062 \cdot 10^3 \pm 1.644 \cdot 10^3$	$69.053 \cdot 10^3 \pm 71.771 \cdot 10^3$	60887	0.9961	3
	Gilt-head sea bream	3-80	$34.607 \cdot 10^3 \pm 2.626 \cdot 10^3$	$139.975 \cdot 10^3 \pm 111.912 \cdot 10^3$	95760	0.9914	3

a: intercept; b: slope; s_a: standard deviation of the intercept; s_b: standard deviation of the slope; $s_{y/x}$: standard deviation of the estimate; R^2: determination coefficient.

Table S6.- Limits of quantification of the method in European sea bass and gilt-head sea bream.

Analyte	Matrix	LOQ_{method} (ng/g)
Sulfadiazine	European sea bass	21.8
	Gilt-head sea bream	25.7
Diaveridine	European sea bass	26.8
	Gilt-head sea bream	26.4
Trimethoprim	European sea bass	27.1
	Gilt-head sea bream	26.5
Marbofloxacin	European sea bass	111.9
	Gilt-head sea bream	96.9
Enrofloxacin	European sea bass	68.6
	Gilt-head sea bream	62.6
Difloxacin	European sea bass	61.1
	Gilt-head sea bream	58.8
Sulfamethoxazole	European sea bass	20.5
	Gilt-head sea bream	23.8
Oxalinic acid	European sea bass	30.4
	Gilt-head sea bream	30.3
Tilmicosin	European sea bass	34.6
	Gilt-head sea bream	30.8
Flumequine	European sea bass	35.8
	Gilt-head sea bream	32.6

Table S7.- Concentrations of the different target analytes found in European sea bass (n=10) and gilt-head sea bream (n=10) samples.

**Analyte**	**Gilt-head sea bream 1**	**Gilt-head sea bream 2**	**Gilt-head sea bream 3**	**Gilt-head sea bream 4**	**Gilt-head sea bream 5**	**Gilt-head sea bream 6**	**Gilt-head sea bream 7**	**Gilt-head sea bream 8**	**Gilt-head sea bream 9**	**Gilt-head sea bream 10**
Sulfadiazine	ND	ND	ND	ND	ND	ND	ND	ND	ND	ND
Diaveridine	ND	ND	ND	ND	ND	ND	ND	ND	ND	ND
Trimethoprim	ND	ND	ND	ND	ND	ND	ND	ND	ND	ND
Marbofloxacin	ND	ND	ND	ND	ND	ND	ND	ND	ND	ND
Enrofloxacin	ND	ND	ND	ND	ND	ND	ND	ND	ND	ND
Difloxacin	ND	ND	ND	ND	ND	ND	ND	ND	ND	ND
Sulfamethoxazole	ND	ND	ND	ND	ND	ND	ND	ND	ND	ND
Oxolinic acid	ND	ND	ND	ND	ND	ND	ND	ND	ND	ND
Tilmicosin	ND	ND	ND	ND	ND	ND	ND	ND	ND	ND
Flumequine	ND	ND	ND	ND	ND	ND	ND	ND	ND	ND
**Analyte**	**European sea bass 1**	**European sea bass 2**	**European sea bass 3**	**European sea bass 4**	**European sea bass 5**	**European sea bass 6**	**European sea bass 7**	**European sea bass 8**	**European sea bass 9**	**European sea bass 10**
Sulfadiazine	ND	ND	ND	ND	ND	ND	ND	ND	ND	ND
Diaveridine	ND	ND	ND	ND	ND	ND	ND	ND	ND	ND
Trimethoprim	ND	ND	ND	ND	ND	ND	ND	ND	ND	ND
Marbofloxacin	ND	ND	ND	ND	ND	ND	ND	ND	ND	ND
Enrofloxacin	ND	ND	ND	ND	ND	ND	ND	ND	ND	ND
Difloxacin	ND	ND	ND	ND	ND	ND	ND	ND	ND	ND
Sulfamethoxazole	ND	ND	ND	ND	ND	ND	ND	ND	ND	ND
Oxolinic acid	<LOQ	<LOQ	<LOQ	<LOQ	<LOQ	<LOQ	<LOQ	<LOQ	<LOQ	<LOQ
Tilmicosin	ND	ND	ND	ND	ND	ND	ND	ND	<LOQ	<LOQ
Flumequine	ND	ND	ND	ND	ND	ND	ND	ND	ND	ND

LOQ: limit of quantification of the method; ND: not detected
